# Comment on Maglione et al. Changes in Respiratory Viruses’ Activity in Children During the COVID-19 Pandemic: A Systematic Review. *J. Clin. Med.* 2025, *14*, 1387

**DOI:** 10.3390/jcm14082536

**Published:** 2025-04-08

**Authors:** Marco Del Riccio, Vieri Lastrucci

**Affiliations:** 1 Department of Health Sciences, University of Florence, 50134 Florence, Italy; 2 Epidemiology Unit, Meyer Children’s Hospital IRCCS, 50139 Florence, Italy

We read with great interest the recently published systematic review by Maglione et al. in the Journal of Clinical Medicine [1]. Their systematic review provides insight into the circulation trends of respiratory viruses throughout the COVID-19 pandemic in children, a relevant and current subject matter for public health [2,3]. The authors have conducted an extensive and meticulous analysis, and we commend their efforts in synthesizing the existing literature. In fact, due to the significant impact of pandemic control measures on respiratory infections, this is still a topic that deserves close monitoring and further research [4,5].

We would like to take this opportunity to bring attention to our study, “Seasonality and Severity of Respiratory Syncytial Virus During the COVID-19 Pandemic: A Dynamic Cohort Study”, published in the International Journal of Infectious Diseases. This manuscript was accepted in August 2024 and made available online as “in press” in early September, indexed in major databases such as PubMed and Scopus. As the review by Maglione et al. included studies up to November 2024, it is possible that this study, though publicly available during the search period, was inadvertently missed [6]. Our research aligns closely with the topics discussed in the review and provides more data that would have complemented the current findings: since our article discusses respiratory syncytial virus (RSV) seasonality, epidemiological changes, and disease severity after pandemic mitigation measures, we feel that its inclusion would have provided a broader view of this significant topic.

The aim of our study was to assess whether the lifting of COVID-19 containment measures led to changes in RSV epidemiology in children, particularly in terms of seasonality, age distribution, and disease severity. Our work, which was based on a large dynamic cohort study in Tuscany, Italy, provides important evidence in this area, and this specific design allowed for continuous monitoring of population-level trends across multiple seasons, helping our group in assessing shifts in age-related burden and timing of epidemics. Specifically, we observed the following:

- A significant increase in RSV-associated hospitalizations following the lifting of pandemic restrictions, with an incidence rate 3.6 times higher in the 2022–2023 season, compared to pre-pandemic seasons;
- A change in RSV seasonality, with earlier outbreaks and shorter epidemics, as reported by Maglione et al. described in other regions by other studies [1,3];
- A higher burden of severe RSV cases among older children (≥12 months), a phenomenon that we attribute to an immunity debt effect, which aligns with the broader trends discussed in the systematic review.

Bechini and colleagues reported similar trends in RSV seasonality and severity by analyzing RSV-associated hospitalization in Italian children, highlighting the importance of implementing preventive strategies such as maternal immunization and monoclonal antibodies [7]. These findings reinforce and extend upon the observations made in the systematic review by Maglione et al., reinforcing the public health importance of RSV surveillance and the potential impact of new RSV preventive strategies. Given that RSV seasonality and severity appear to be undergoing a post-pandemic shift, we believe continued research and discussion in this area is and will be crucial for future public health planning.
